# Stability of the cancer target DDIAS is regulated by the CHIP/HSP70 pathway in lung cancer cells

**DOI:** 10.1038/cddis.2016.488

**Published:** 2017-01-12

**Authors:** Kyoung-Jae Won, Joo-Young Im, Bo-Kyung Kim, Hyun Seung Ban, Young-Jin Jung, Kyeong Eun Jung, Misun Won

**Affiliations:** 1 Personalized Genomic Medicine Research Center, KRIBB, Daejeon 305-806, Korea; 2 Functional Genomics, University of Science and Technology, Daejeon 305-701, Korea; 3 Metabolic Regulation Research Center, KRIBB, Daejeon 305-806, Korea; 4 Oligo Team, ST Pharm. Co., LTD, Sihwa Industrial Complex 1, Kyunggido 429-848, Korea

## Abstract

DNA damage-induced apoptosis suppressor (DDIAS) rescues lung cancer cells from apoptosis in response to DNA damage. DDIAS is transcriptionally activated by NFATc1 and EGF-mediated ERK5/MEF2B, leading to cisplatin resistance and cell invasion. Therefore, DDIAS is suggested as a therapeutic target for lung cancer. Here, we report that DDIAS stability is regulated by E3 U-box ubiquitin ligase carboxyl terminus of HSP70-interacting protein (CHIP)-mediated proteasomal degradation. We first isolated CHIP as an interacting partner of DDIAS by yeast two-hybrid screening. CHIP physically associated with both the N- and C-terminal regions of DDIAS, targeting it for proteasomal degradation and reducing the DDIAS half-life. CHIP overexpression analyses indicated that the tetratrico peptide repeat (TPR) domain and the U-box are required for DDIAS ubiquitination. It is likely that HSP70-bound DDIAS is recruited to the CHIP E3 ligase via the TPR domain, suggesting DDIAS as a client protein of HSP70. In addition, CHIP overexpression in lung cancer cells expressing high DDIAS levels induced significant growth inhibition by enhancing DDIAS degradation. Furthermore, simultaneous CHIP overexpression and DNA damage agent treatment caused a substantial increase in the apoptosis of lung cancer cells. Taken together, these findings indicate that the stability of the DDIAS protein is regulated by CHIP/HSP70-mediated proteasomal degradation and that CHIP overexpression stimulates the apoptosis of lung cancer cells in response to DNA-damaging agents.

Protein turnover is an essential process in regulating cellular function, which prevents the accumulation of unnecessary proteins. E3 U-box ubiquitin ligase carboxyl terminus of HSP70-interacting protein (CHIP) has crucial roles in maintaining the steady-state levels of a number of target proteins via the ubiquitin–proteasome system.^1, 2, 3, 4^ CHIP contains a U-box for interaction with the E2 ubiquitin conjugating enzyme and directs the ubiquitination of substrates and a tetratrico peptide repeat region (TPR) domain for interaction with the chaperones heat shock protein 70 (HSP70) or HSP90. CHIP is involved not only in homeostatic regulation under resting conditions but also in various stress-activated signaling pathways.^4, 5^ Therefore, CHIP is associated with diseases such as cancer, neurological disorders, cardiac disease and bone metabolism.^4^ CHIP has been considered as a tumor suppressor because it negatively regulates oncoproteins such as Akt, hypoxia inducible factor 1*α* (HIF-1*α*), BCR-ABL, human telomerase reverse transcriptase (hTERT) and c-Myc.^6, 7, 8^ In accordance with this, CHIP expression is significantly downregulated in a number of cancers.^9^ In contrast, CHIP also functions as an oncogene by targeting phosphatase and tensin homolog (PTEN) for proteasomal degradation in prostate cancer cells.^10^ CHIP-null mice are temperature-sensitive and develop multi-organ apoptosis after heat shock, indicating the role of CHIP in the response to stress.^11^ In addition, CHIP suppresses cancer stem cell properties in breast cancer cells.^12^ CHIP is also involved in the degradation of proteins during single-strand break repair/base excision repair.^13, 14, 15^

Recently, DNA damage-induced apoptosis suppressor (DDIAS) has been suggested as a promising therapeutic target in several cancers.^16, 17, 18, 19^ DDIAS is highly expressed in lung cancer and hepatocellular carcinoma cells and promotes cellular proliferation, colony formation, cellular migration and *in vivo* tumorigenicity.^16, 17, 18^ We have previously revealed that DDIAS knockdown induces apoptosis in cancer but not normal cells.^16^ In addition, we demonstrated that DDIAS knockdown concomitant with exposure to DNA-damaging agents enhances cancer cell death synergistically. In contrast, DDIAS overexpression restores the induction of lung cancer cell apoptosis by DNA damage agents, indicating that DDIAS functions as a DNA damage-induced suppressor of apoptosis.^16, 19^ DDIAS is transcriptionally activated by nuclear factor of activated T cells (NFAT2).^18, 19^ In response to epidermal growth factor (EGF), the transcription of DDIAS is also activated via ERK5/MEF2B signaling, thereby promoting cell invasion by *β*-catenin accumulation.^20^ In addition to inhibiting DDIAS transcription, targeting DDIAS degradation mechanisms might also be crucial to suppress cancer cell growth. However, little is known regarding the DDIAS protein degradation process. Therefore, we investigated the mechanism that regulates DDIAS protein turnover.

Here, we report that DDIAS protein turnover is regulated by CHIP-mediated proteasomal degradation. We also demonstrate that CHIP overexpression is linked to the growth inhibition of lung cancer cells by enhancing DDIAS degradation.

## Results

### Identification of CHIP as a regulator of DDIAS proteasomal degradation

To search for proteins that interacted with DDIAS, we performed yeast two-hybrid screening. The fusion protein of the DNA-binding domain in the GAL4 transcription factor with the C-terminal region of DDIAS (aa 784–998) was used as bait because the remaining DDIAS protein sequence exhibited false positives owing to DNA-binding activity (Supplementary Figure S1). A HeLa cell cDNA library fused with the activation domain of GAL4 was used as the prey. From this analysis, we identified 23 genes encoding potential binding partners for DDIAS including *CHIP* (*STUB1*, NM_005861), *EFEMP1* (NM_001039349), *ECM1* (NM_001202858), *FBLN1* (NM_001996/NM_006486), *CBY1* (NM_001002880), *MIF4GD* (NM_020679), *ACTN4* (NM_004924), *ACTN1* (NM_001130005), *MAF1* (NM_032272), *PLSCR1* (NM_021105) and *ACLY* (NM_198830).

**Figure 1:**
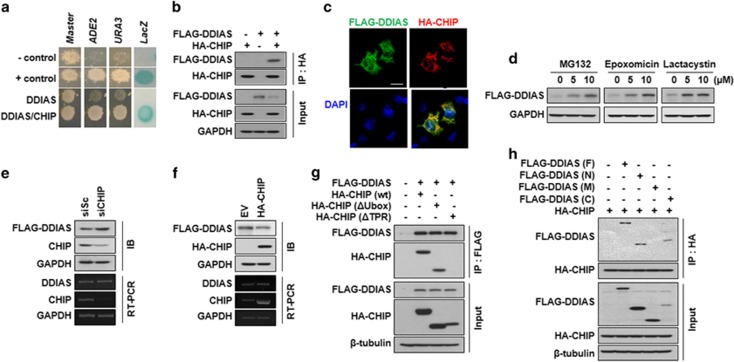
Identification of E3 ligase CHIP for proteasomal degradation of DDIAS. (**a**) Identification of E3 ligase CHIP as a DDIAS-interacting partner. Yeast two-hybrid analysis was performed using GAL4 DNA-binding domain (DB)-fused DDIAS (aa 784–998) and GAL4 transcriptional activation domain (AD)-CHIP. Three independent reporters, beta-galactosidase (SD-LW), ADE2 (SD-LWA) and URA3 (SD-LWU) were used. As a negative control, pGBKT7 and pGADT7 were used. Dimerization of PTB using pGBKT7-PTB and pGADT7-PTB was used as a positive control. (**b**) Interaction of DDIAS and CHIP. Co-IP was performed using cell lysates obtained from HEK293T cells co-transfected with FLAG-DDIAS and HA-CHIP plasmids. (**c**) Co-localization of FLAG-DDIAS and HA-CHIP. Immunocytochemical staining was performed in cells co-transfected with FLAG-DDIAS and HA-CHIP plasmids. FLAG-DDIAS and HA-CHIP were detected by FITC- and rhodamine-conjugated antibodies, respectively. Scale bar indicates 20 *μ*m. (**d**) Effect of proteasome inhibitors on the degradation of FLAG-DDIAS. HEK293T cells transfected with FLAG-DDIAS were treated with proteasome inhibitors MG132, epoxomicin and lactacystin. Changes in FLAG-DDIAS protein levels were detected. (**e** and **f**) Effect of CHIP expression on the degradation of FLAG-DDIAS. FLAG-DDIAS expression level was examined in cell lysates prepared from HEK293T cells after overexpression or knockdown of CHIP using HA-CHIP plasmid or siCHIP, respectively. (**g**) Interaction of FLAG-DDIAS and HA-CHIP domain. Cell lysates were prepared from cells co-transfected with FLAG-DDIAS and domain-deleted HA-CHIP (△Ubox or △TPR) vectors and used for Co-IP with anti-FLAG antibody. (**h**) DDIAS domain analysis for CHIP binding in HEK293T cells. The cell lysates obtained from cells expressing polypeptides of F (aa 1–998), N (aa 1–400), M (aa 401–600) and C (aa 601–998) domains were used for Co-IP with anti-HA antibody

To investigate the mechanism of DDIAS turnover in cells, we selected the E3 ubiquitin ligase CHIP for further study because CHIP promotes the ubiquitin ligation/chain elongation-mediated proteasomal degradation of target genes in cancer cells.^12, 15, 21^ First, we demonstrated that DDIAS interacted with CHIP using three reporters (URA3, lacZ and ADE2) in yeast. Specifically, the transformant expressing DDIAS (aa 784–998) and CHIP grew well on the plate lacking adenine (SD-ALW) or uracil (SD-ULW), whereas negative control cells expressing only DDIAS or CHIP did not (Figure 1a). Similarly, the transformant expressing both DDIAS (aa 784–998) and CHIP also exhibited *β*-galactosidase activity, whereas negative controls did not. The dimerization of polypyrimidine tract-binding protein (PTB) in cells containing both pGBKT7-*PTB* and pGADT7-*PTB* served as a positive control.

Next, we assessed the physical interaction of DDIAS with CHIP in mammalian cells. Coimmunoprecipitation (Co-IP) assays clearly showed that FLAG-DDIAS interacted with HA-CHIP *in vivo* (Figure 1b). Upon immunocytochemical staining, DDIAS co-localized with CHIP primarily in the cytoplasm of cells (Figure 1c). As CHIP promotes proteasomal degradation through the ligation of ubiquitin to its target proteins, we tested whether DDIAS undergoes degradation via ubiquitin-mediated proteasomal degradation in HEK293T cells. In the presence of the proteasomal inhibitors MG132, epoxomicin or lactacystin, DDIAS protein levels greatly increased (Figure 1d), suggesting that DDIAS normally undergoes ubiquitin-mediated proteasomal degradation. We next evaluated whether CHIP was associated with the degradation of DDIAS. CHIP genetic knockdown significantly increased the FLAG-DDIAS protein level but not the *DDIAS* mRNA level (Figure 1e). In contrast, HA-CHIP overexpression resulted in a reduction of the FLAG-DDIAS protein level, whereas again no alteration was observed in the *DDIAS* mRNA level (Figure 1f). This result suggests that the E3 ligase CHIP interacted with DDIAS in the cytoplasm, leading to its proteasomal degradation.

To determine which domain of CHIP interacted with DDIAS, we utilized Co-IP in HEK293T cells (Figure 1g). No DDIAS binding was observed following deletion of the CHIP TPR domain, whereas deletion of the U-box demonstrated DDIAS binding. This indicated that the TPR domain was required for the binding of CHIP to DDIAS. We then examined which domain of FLAG-DDIAS interacted with HA-CHIP *in vivo* using Co-IP (Figure 1h). The domain analysis of DDIAS demonstrated that polypeptides of both the N (aa 1–400) and C (aa 601–998) termini but not of M (aa 401–600) interacted with the HA-CHIP protein (Figure 1h). Taken together, these results imply that DDIAS turnover is regulated by CHIP-mediated proteasomal degradation through binding of the TPR domain of CHIP to the N- and C-terminal regions of DDIAS in HEK293T cells.

### HSP70 recruits DDIAS to the CHIP E3 ligase, whereas CHIP promotes the ubiquitination of DDIAS

**Figure 2:**
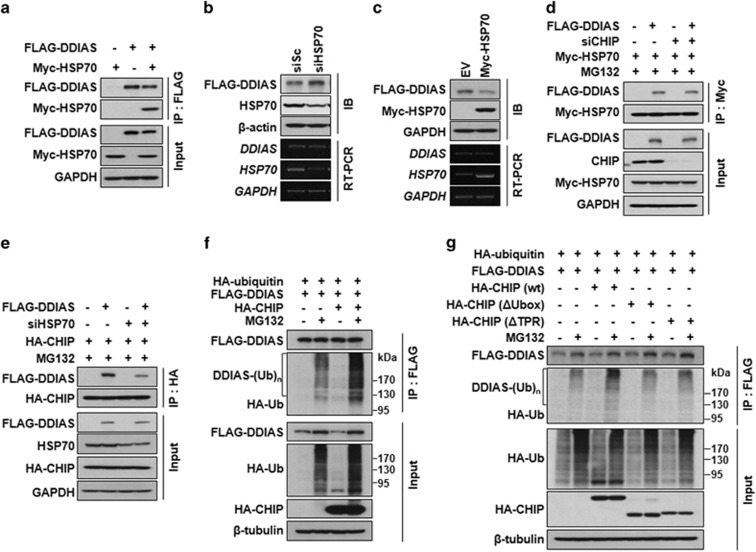
DDIAS is a client of HSP70 for CHIP-mediated proteasomal degradation. (**a**) Interaction of DDIAS with HSP70. Co-IP was performed using cell lysates obtained from HEK293T cells co-transfected with FLAG-DDIAS and Myc-HSP70. (**b** and **c**) Effect of HSP70 on the degradation of FLAG-DDIAS. FLAG-DDIAS expression level was examined in cell lysates prepared from HEK293T cells after knockdown using siHSP70 (**b**) or overexpression using Myc-HSP70 (**c**). (**d**) Effect of CHIP knockdown on the interaction between HSP70 and DDIAS. HEK293T cells co-transfected with FLAG-DDIAS and Myc-HSP70 vectors were treated with siCHIP. Co-IP was performed using anti-Myc antibody. (**e**) Effect of HSP70 knockdwon on the interaction between CHIP and DDIAS. HEK293T cells co-transfected with FLAG-DDIAS, and HA-CHIP were treated with siHSP70. Co-IP was performed using anti-HA antibody. (**f**) Ubiquitination of DDIAS by CHIP. Cell lysates were prepared from cells co-transfected with HA-ubiquitin and FLAG-DDIAS vectors with or without HA-CHIP expression in the presence of MG132 (10 *μ*M). Ubiquitinated FLAG-DDIAS was detected by immunoprecipitation using anti-FLAG antibody. (**g**) Effect of CHIP domain on the ubiquitination of FLAG-DDIAS. FLAG-DDIAS were co-expressed with CHIP△Ubox (aa 1–195) or CHIP△TPR (aa 128–303) in HEJK293T cells followed by treatment with MG132 (10 *μ*M). Cell lysates were immunoprecipitated with anti-FLAG antibody beads, and ubiquitinated DDIAS was detected

CHIP interacts with chaperone HSP70 to affect the ubiquitination of target proteins. Because DDIAS interacted with the TPR domain responsible for HSP70-binding, we asked whether HSP70 is involved in the degradation of DDIAS as a chaperone in HEK293T cells. FLAG-DDIAS clearly interacted with HSP70 *in vivo* as shown by a Co-IP assay (Figure 2a). In addition, HSP70 knockdown significantly increased FLAG-DDIAS protein level without alteration of *DDIAS* mRNA level (Figure 2b). Conversely, Myc-HSP70 overexpression reduced level of DDIAS, suggesting that DDIAS degradation is regulated by CHIP in an HSP70-dependent manner (Figure 2c).

To investigate the function of HSP70 in the CHIP-mediated proteasomal degradation of DDIAS, we determined the binding strength between CHIP and DDIAS in the absence of HSP70. Notably, CHIP knockdown did not significantly affect the interaction between DDIAS and HSP70 in the presence of MG132 (Figure 2d). However, HSP70 knockdown dramatically weakened the interaction between CHIP and FLAG-DDIAS, suggesting that HSP70 mediates the binding of DDIAS to CHIP (Figure 2e). Accordingly, it is likely that HSP70 recruits DDIAS as its client protein for binding to CHIP, which allows CHIP to associate physically with the N (aa 1–400) and C (aa 601–998) terminal regions in DDIAS.

Next, we examined whether the DDIAS protein was polyubiquitinated for proteasomal degradation by CHIP in HEK293T cells. The polyubiquitination of FLAG-DDIAS was observed in the presence of MG132, whereas it was barely detected in the absence of MG132. Furthermore, DDIAS polyubiquitination significantly increased in the cells expressing HA-CHIP compared with those not expressing HA-CHIP (Figure 2f). However, a significant ub-signal is still observed even in the absence of HA-CHIP, presumably due to the presence of endogenous CHIP. This result indicated that CHIP mediates the ubiquitination of DDIAS for proteasomal degradation. We then assessed which domain of CHIP was important for DDIAS ubiquitination (Figure 2g). Overexpression of CHIP with deletions of the TPR (CHIP-ΔTPR) or the U-box (CHIP-ΔUbox) demonstrated significant decreases in DDIAS polyubiquitination, indicating that both domains are required for DDIAS ubiquitination (Figure 2g). This result also supports that HSP70-bound DDIAS is recruited to the CHIP E3 ligase via TPR and then ubiquitinated by the CHIP E3 ligase via the U-box.

### CHIP overexpression increases DDIAS turnover in HEK293T cells

**Figure 3:**
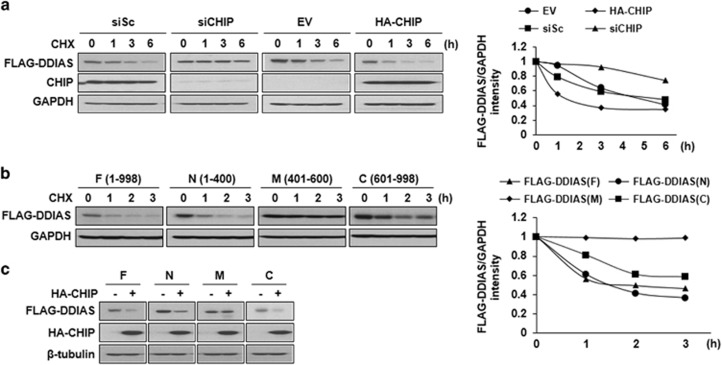
Domain-specific degradation of DDIAS by the CHIP-mediated proteasomal degradation. (**a**) CHIP regulates stability of FLAG-DDIAS. Stability of FLAG-DDIAS was examined by CHIP knockdown and CHIP overexpression using CHIP siRNA and HA-CHIP vector, respectively in HEK293T cells. Changes in FLAG-DDIAS levels were examined in the presence of cycloheximide (CHX, 20 *μ*M) for the indicated time period. (**b**) Stabilities of DDIAS domains. The expression levels of DDIAS domains (N, M and C) were determined in the presence of CHX for the indicated time period. (**c**) Effect of HA-CHIP overexpression on the expression level of DDIAS domain. The expression of DDIAS domains (F, N, M and C) was determined in HEK293T cells transiently expressing HA-CHIP

As CHIP is involved in the proteasomal degradation of DDIAS, we assessed the effect of CHIP on the stability of the DDIAS in HEK293T cells (Figure 3a). CHIP knockdown increased the half-life of FLAG-DDIAS from approximately 3 to >6 h. In contrast, the overexpression of HA-CHIP reduced the half-life of FLAG-DDIAS to approximately 1.2 h. This result indicates that CHIP has a crucial role in regulating DDIAS turnover in cells. Therefore, we postulated that the binding of CHIP to the individual DDIAS domains correlated with their respective stabilities. When the stability of each domain was examined, the CHIP binding, N (aa 1–400) and C (aa 601–998) regions were found to be more unstable compared with the unbound and M (aa 401–600) regions (Figure 3b). Consistent with this, HA-CHIP overexpression significantly reduced the expression of both the N- (aa 1–400) and C- (aa 601–998) terminal regions of the DDIAS protein but not of M (aa 401–600; Figure 3c). It is likely that the high turnover rate of both the N and C domains is associated with CHIP-mediated degradation. This observation strongly suggests that CHIP increases turnover of the DDIAS protein by targeting both the N- and C-terminal regions.

### CHIP levels correlate with DDIAS instability and DDIAS depletion-induced growth inhibition in lung cancer cells

**Figure 4:**
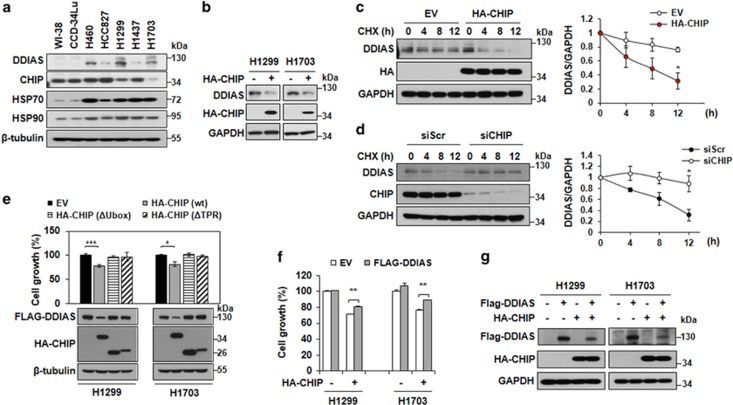
DDIAS level is regulated by CHIP expression in lung cancer cells. (**a**) Expression of CHIP and DDIAS in lung cell lines. Normal lung fibroblast cells, WI-38 and CCD-34 LU, and lung cancer cells, NCI-H460, HCC-827, NCI-H1299, NCI-H1437 and NCI-H1703, were used. Cell lysates of indicated cell lines were immunoblotted to detect expression of DDIAS, CHIP, HSP70 and HSP90. (**b**) CHIP overexpression decreased endogenous DDIAS protein level in NSCLC. H1299 and H1703 cells were transfected with HA-CHIP for 48 h and lysed. Endogenous DDIAS protein level was examined using anti-DDIAS, anti-HA and anti-GAPDH. (**c**) Stabilities of DDIAS by HA-CHIP overexpression in NCI-H1299. DDIAS stability was determined in HA-CHIP-overexpressed NCI-H1299 cells in the presence of CHX (20 *μ*M). (**d**) The CHIP knockdown effect on DDIAS stability in NCI-H1437 cells. DDIAS stability was determined in cells transfected with CHIP siRNA. (**e**) The effect of CHIP overexpression on the growth of NCI-H1299 and NCI-H1703 cells. Growth inhibition by CHIP overexpression was determined by an SRB assay. The growth of NCI-H1299 or NCI-H1703 cells transiently expressing HA-CHIP or CHIP mutants was also compared. The values represent the mean ± SEM of three independent experiments with triplicated measurements. *P<0.05, ***P<0.005 vs Con. (**f**) The effect of Flag-DDIAS overexpression on the growth of NCI-H1299 and NCI-H1703 cells. The effect of Flag-DDIAS overexpression on the growth inhibition by HA-CHIP overexpression was determined by an SRB assay. The values represent the mean ± SEM of three independent experiments with triplicate measurements. **P<0.01 vs Con. (**g**) Western blot analysis of cells overexpressing FLAG-DDIAS and/or HA-CHIP

We previously demonstrated that DDIAS is highly expressed in various cancer tissues, although tissue-specific DDIAS expression in normal tissues is observed.^16^ To evaluate the relevance of DDIAS to cancerous properties, we determined the level of DDIAS protein in lung cancer cells. We found that DDIAS expression was relatively high and varied in lung cancer cells, although it was detected at very low levels in lung fibroblast cells, WI-38 and CCD-34Lu cells (Figure 4a). The expression of both HSP70 and HSP90 was increased in lung cancer cells compared with normal lung fibroblast cells. First, we confirmed endogenous DDIAS level is reduced by CHIP overexpression in NCI-H1299 and NCI-H1703 (Figure 4b). In NCI-H1299 cells expressing relatively low CHIP, endogenous DDIAS was relatively stable with a long half-life of >12 h (Figure 4c). We then tested whether the forced overexpression of HA-CHIP affected the extended DDIAS stability in NCI-H1299 cells HA-CHIP overexpression significantly decreased the turnover of DDIAS yielding a half-life of approximately 8 h (Figure 4c). On the contrary, half-life of DDIAS in NCI-H1437 expressing high CHIP was about 9 h, which was increased dramatically to more than 12 h by CHIP knockdown (Figure 4d). This result implicates that CHIP expression correlates with DDIAS instability in lung cancer cells.

We previously reported that DDIAS downregulation suppresses the growth of lung cancer cells.^16^ Therefore, we assessed whether HA-CHIP overexpression affects the growth of NCI-H1299 and NCI-H1703 cells through DDIAS degradation. Our results indicated that HA-CHIP overexpression induced DDIAS downregulation and the growth inhibition of NCI-H1299 and NCI-H1703 cells (Figure 4e). In contrast, the overexpression of Flag-DDIAS partially protected NCI-H1299 and NCI-H1703 cells from the growth inhibition by HA-CHIP overexpression (Figure 4f). We also confirmed that Flag-DDIAS was still present even with HA-CHIP overexpression (Figure 4g). These findings suggest that CHIP overexpression in lung cancer cells induces growth inhibition by enhancing DDIAS degradation.

### CHIP overexpression plus DNA damage substantially enhances the growth inhibition of cancer cells

**Figure 5:**
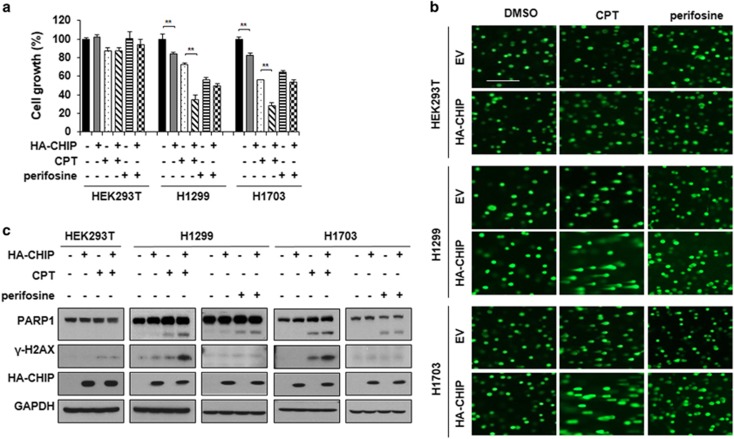
Simultaneous CHIP overexpression and DNA damage substantially enhances the growth inhibition of cancer cells. (**a**) Effects of CHIP overexpression on the growth inhibition of cells treated with CPT. Growth inhibition of cancer cells by transient overexpression of CHIP was determined in the presence of CPT. Perifosine was used as a negative control. The values represent the mean ± SEM of three independent experiments with triplicate measurements. **P<0.01 vs Con. (**b**) Comet assay of cells overexpressing HA-CHIP on CPT exposure. The comet assay was performed to visualize DNA damage of the cells transfected with HA-CHIP on CPT exposure. Perifosine was used as a negative control. Scale bar indicates 200 *μ*m. (**c**) Apoptosis analysis of cells transiently overexpressing CHIP on CPT exposure. Immunoblot analysis was performed using antibody of PARP1and γ-H2AX

In an earlier study, we demonstrated that DDIAS knockdown induced a synergistic effect on growth inhibition with concomitant treatment by a DNA damage-inducing agent such as CPT and cisplatin.^16^ Therefore, we tested whether CHIP overexpression enhances the growth inhibition of NCI-H1299 and NCI-H1703 cells in the presence of CPT or of perifosine, which acts to induce apoptosis through Akt inhibition, as a negative control (Figure 5a). The growth of HEK293T cells was not significantly affected by either CPT or perifosine at the concentrations used. HA-CHIP overexpression also had no effect on the growth of HEK293T cells. However, HA-CHIP overexpression with simultaneous CPT treatment substantially augmented the growth inhibitory effect on NCI-H1703 and NCI-H1299 (Figure 5a). In contrast, perifosine did not significantly affect the growth inhibition caused by CHIP overexpression in the cells with low endogenous CHIP levels.

Previously, we also suggested that DDIAS function is associated with DNA repair in the presence of DNA-damaging agents. Specifically, simultaneous DDIAS depletion and treatment with a DNA damage-inducing agent caused synergistic effects on DNA damage and the growth inhibition of A549 cells.^16^ Therefore, we asked whether HA-CHIP overexpression markedly impacts DNA damage. In comet assays, CPT treatment of cells overexpressing HA-CHIP showed a synergistic effect on DNA damage with longer-tailed comets in NCI-H1299 and NCI-H1703 cells (Figure 5b). However, concomitant treatment of these cells to both perifosine and CHIP overexpression did not yield this effect. Furthermore, HEK293T cells showed no detectable change in comet lengths. In addition, western blot analysis also revealed that HA-CHIP overexpression caused a significant effect on the apoptosis of both NCI-H1299 and NCI-H1703 cells upon CPT exposure, resulting in the increase of PARP-1 cleavage and γH2AX in these cells (Figure 5c). This result suggests that DDIAS depletion mediated by CHIP overexpression and the simultaneous induction of DNA damage caused significant cell death only in cancer cells expressing high levels of DDIAS.

## Discussion

To examine the mechanism underlying proteasomal degradation of DDIAS, we first performed a yeast two-hybrid assay and isolated E3 ligase CHIP as a potential DDIAS-interacting partner. Additional DDIAS-interacting proteins identified included EFEMP1,^22^ ECM1,^23^ CBY1,^24^ PLSCR1 (ref. 25) and MIF4GD,^26^ which are involved in cell survival, proliferation, metastasis, invasion, resistance to apoptosis and cell adhesion.

We next revealed that CHIP knockdown decreased the turnover rate of exogenous DDIAS, increasing its stability in HEK293T cells. Conversely, CHIP overexpression significantly increased DDIAS turnover, decreasing its stability. CHIP targeted the N- and C-terminal regions of DDIAS for its ubiquitination and proteasomal degradation. We also demonstrated that HSP70 recruited DDIAS for binding to CHIP, identifying DDIAS as a client protein of HSP70. Therefore, we suggest that DDIAS stability is regulated as a novel target of the CHIP/HSP70-mediated ubiquitin–proteasomal degradation pathway.

As CHIP is associated with chaperones HSP70 or HSP90 for the ubiquitination of target proteins,^27^ we examined the involvement of HSP90 in the ubiquitination-mediated degradation of DDIAS. Notably, HSP90 knockdown led to reduced DDIAS protein level (Supplementary Figure S2), suggesting that HSP90 might not function as a chaperone for DDIAS ubiquitination. However, the findings clearly demonstrated that HSP70 was solely involved in CHIP-mediated proteasomal degradation of DDIAS.

DDIAS expression levels differ between human tissues, and DDIAS knockdown does not noticeably affect the growth of either WI-38 or HEK293T cells.^16, 19^ Furthermore, we found that HA-CHIP overexpression did not cause either DNA damage or growth inhibition in HEK293T cells, suggesting that DDIAS downregulation may not seriously compromise normal cells. In addition, we revealed that CHIP expression is lower in NCI-H1299 and NCI-H1703 cells, in which endogenous DDIAS is more stable with >12 h half-life, compared with the 9 h half-life of DDIAS in NCI-H1437 cells exhibiting high CHIP expression. However, CHIP overexpression in NCI-H1299 led to increased turnover of DDIAS, resulting in a reduction of DDIAS half-life from >12 to 8 h. In contrast, CHIP knockdown in NCI-H1437 resulted in increased half-life from 9 to >12 h, indicating correlation of CHIP expression level with DDIAS turnover rate. Furthermore, we have shown that CHIP overexpression induced significant growth inhibition of NCI-H1299 and NCI-H1703 cells through DDIAS downregulation. Thus, we suggest that cells expressing low CHIP level are associated with DDIAS-dependent growth inhibition and are likely sensitive to DDIAS depletion, as shown for NCI-H1299 and NCI-H1703 cells. In addition, DDIAS overexpression suppressed growth inhibition caused by CHIP overexpression in NCI-H1299 or NCI-H1703 cells, suggesting CHIP-dependent DDIAS instability is directly linked with the growth of cancer cells.

Previously, we showed that simultaneous DDIAS depletion and DNA damage has synergistic effects on DNA damage and growth inhibition in A549 cells.^16^ Consistent with this, HA-CHIP overexpression with simultaneous CPT treatment resulted in a substantial enhancing effect on the induction of apoptosis in NCI-H1703 and NCI-H1299. CHIP overexpression greatly sensitized the former cells to CPT, resulting in a significant inhibition of cell growth. Furthermore, CPT treatment simultaneous with HA-CHIP overexpression substantially impacted the degree of DNA damage yielding longer-tailed comets in comet assays in NCI-H1299 and NCI-H1703 cells, whereas this effect was not observed following simultaneous perifosine treatment. Notably, DDIAS is also associated with cisplatin resistance in lung cancer cells.^19^ Therefore, it is likely that DDIAS is associated with DNA repair by suppressing DNA damage in the presence of DNA-damaging agents in NCI-H1299 and NCI-H1703 cells. Studies are currently underway to better understand the involvement of DDIAS in DNA repair.

We observed the significant growth recovery by forced DIAAS overexpression in CHIP overexpressing lung cancer cells, suggesting that increase of DDIAS turnover by CHIP overexpression noticeably contributes to growth inhibition of cells. Recently, many reports have demonstrated that CHIP negatively regulates the expression of various genes including *VEGFR2*, *c-Myc* and *PRMT5*, which mediates proliferation, cell migration, invasion and angiogenesis.^6, 9, 28^ Therefore, we cannot exclude the possibility of pleiotropic effects of HA-CHIP overexpression on growth inhibition of lung cancer cells, which might represent a limitation of this study.

Notably, *DDIAS* mRNA expression correlated approximately 73% with NFATc1 protein expression, indicating a crucial role of the transcriptional regulation of this protein.^19^ However, the inverse correlation between DDIAS and CHIP expression implicates CHIP/HSP70-mediated degradation of DDIAS as a crucial regulatory system for DDIAS expression. Although the importance of the role of CHIP in DDIAS expression in cancer cells remains unclear, CHIP-mediated DDIAS instability might cause significant growth inhibition via DNA damage and apoptosis of cancer cells, but not of normal cells.

In summary, we describe the mechanism controlling DDIAS stability by CHIP/HSP70-mediated proteasomal degradation in lung cancer cells. As a client of HSP70, DDIAS is recruited to the E3 ligase CHIP for proteasomal degradation of the DDIAS protein. In NCI-H1703 and NCI-H1299 cells, CHIP overexpression leads to a significant enhancement of apoptotic cells in the presence of DNA-damaging agents.

## Materials and Methods

### Reagents and antibodies

Tween 20, paraformaldehyde, DAPI (4',6-diamidino-2-phenylindole), sulforhodamine B (SRB), cycloheximide (CHX) were purchased from Sigma-Aldrich (St. Louis, MO, USA). Camptothecin (CPT) and perifosine were obtained from Calbiochem (Darmstadt, Germany) and AdooQ Bioscience LLC (Irvine, CA, USA), respectively. MG132, epoxomicin and lactacystin were supplied by Cayman Chemicals (Ann Arbor, MI, USA). Chemicals were dissolved in DMSO or water at 20 mM concentrations. Rabbit polyclonal antibodies against HA, c-Myc and PARP-1, and mouse monoclonal antibody against luciferase were purchased from Santa Cruz Biotechnology (Santa Cruz, CA, USA). Rabbit polyclonal *β*-tubulin antibody was from Abcam (Cambridge, MA, USA). Mouse monoclonal anti-FLAG (M2) and rabbit polyclonal CHIP antibodies were obtained from Sigma-Aldrich. Rabbit polyclonal GAPDH and *β*-actin antibodies and horseradish peroxidase (HRP)-conjugated secondary antibodies were obtained from AbFrontier (Seoul, Korea). Rabbit polyclonal HSP90 antibody was purchased from Enzo Life Sciences (Farmingdale, NY, USA). FITC or rhodamine B-conjugated secondary antibody and agarose were obtained from Santa Cruz Biotechnology. Anti-FLAG M2 affinity gel and anti-HA agarose were obtained from Sigma-Aldrich.

### Cell culture

Human embryonic kidney cells expressing the SV40 large T antigen (HEK293T), lung fibroblast cells (WI-38, CCD-34Lu) and lung carcinoma cells (NCI-H460, HCC-827, NCI-H1299, NCI-H1703, NCI-H1437) were obtained from the American Type Culture Collection (Manassas, VA, USA) or the KRIBB cell line bank (Daejeon, Korea). Cells were cultured in RPMI 1640 or Dulbecco's modified Eagle's medium (DMEM) (Gibco, Grand Island, NY, USA) supplemented with 10% heat-inactivated fetal bovine serum (Gibco), 100 U/ml penicillin and 100 *μ*g/ml streptomycin (Gibco). The cells were maintained in a humidified incubator at 37 °C with 5% CO_2_. For chemical treamtment of cells, CPT (10 *μ*M, 24 h), cycloheximide (CHX, 20 *μ*M) and perefosine (10 *μ*M, 24 h) were used.

### Yeast two-hybrid analysis

Yeast two-hybrid assay was performed as described previously.^29^ DDIAS (784 aa-end of *DDIAS*, 215 aa) was cloned into BamHI/PstI sites of the pGBKL vector containing the DNA-binding domain of GAL4 (GAL4DB) and *LEU2* as a selection marker in yeast. A human HeLa cDNA AD library was used. Yeast PBN204 strain contains three reporters (*URA3*, *lacZ* and *ADE2*) that are under the control of different *GAL* promoters. Yeast transformants of the DDIAS bait (aa 784–998) and Human HeLa cDNA AD library were spread on selection media [SD-leucine, tryptophan, uracil (SD-LWU)] that supports growth of yeasts with bait and prey plasmids, yielding proteins that interact with each other. We tested protein–protein interactions using beta-galactosidase, ADE2 and URA3 reporters. As the negative control, vectors pGBKT7 and pGADT7 were used. Dimerization of polypyrimidine tract-binding protein (PTB) was used as the positive control by generating pGBKT7-*PTB* and pGADT7-*PTB*.

### Reverse transcription polymerase chain reaction

Total RNA was extracted from cells using TRIzol reagent (Invitrogen, Carlsbad, CA, USA) and reverse transcribed into cDNA using the TOPscript cDNA synthesis kit (Enzynomics, Daejeon, Korea) according to the manufacturer's instructions. Equal amounts of cDNA were diluted and amplified by PCR. The following primers were used: *DDIAS* forward, 5′-CAGAAGCCCTATTGTATCTGG-3′ *DDIAS* reverse, 5′-GTGACCAAGCACTTCGAGTTT-3′ *CHIP* forward, 5′-CGAATCGCGAAGAAGAAGCG-3′; *CHIP* reverse, 5′-GGTCAAAATGACCCACACGC-3′; *HSP70* forward, 5′-GCCTACTTCAACGACTCGCA-3′; *HSP70* reverse, 5′-AGTCGATGCCCTCAAACAGG-3′ 5′-*GAPDH* forward, 5′-TCATGACCACAGTCCATGCC-3′ and *GAPDH* reverse, 5′-TCCACCACCCTGTTGCTGTA-3′.

### Gene knockdown

DDIAS and scrambled siRNAs were synthesized by ST Pharm Co., Ltd (Siheung-si, Korea). The sequences of siRNA were used as follows: *DDIAS*, 5′-CUGUAACCCAGGCAGAUGUdTdT-3′ siScramble, 5′-CCUACGCCACCAAUUUCGUdTdT-3′. Non-Targeting (D-001206-14), *HSP90AA1*(M-005186-02), *HSPA1A*(M-005168-01) and *CHIP*(M-007201-02) from siGENOME SMARTpool were purchased from Dharmacon Inc. (Lafayette, CO, USA). Transfection was performed using Lipofectamine 2000 (Invitrogen, Carlsbad, CA, USA) or DharmaFECT 1 (Lafayette, CO, USA) according to the manufacturer's instructions.

### SRB assay

Cell cytotoxicity was determined with the SRB assay as described previously.^19^ Briefly, the cells were fixed with 4% formaldehyde (Sigma-Aldrich) and stained with SRB solution. The SRB dye bound to the cell matrix was eluted with elution buffer and quantified at 530 nm on a Molecular Devices EMax system (San Diego, CA, USA).

### Gene cloning and transient overexpression in cells

*DDIAS* (1–998 amino acids) and *HSP70* (1-641 amino acids) cDNA clones were obtained from the Korea Human Gene Bank, Medical Genomics Research center, KRIBB, Korea. *DDIAS* Full-length F (aa 1–998) and domain N (aa 1–400), M (aa 401–600) and C (aa 601–998) were amplified by PCR and cloned into the p3xFLAG-CMV vector (Sigma-Aldrich) to create the p3xFLAG-CMV-*DDIAS*. *HSP70*-wt (1-641 amino acids) clone was inserted into pcDNA3 vector with Myc tag (Invitrogen). The coding region of CHIP was obtained by PCR-amplification of HEK293T cDNA. HA-*CHIP*-wt (1-303 amino acids), HA-*CHIP*-ΔTPR (128–303 amino acids) and HA-*CHIP*-ΔUbox (1–195 amino acids) were amplified by PCR and inserted into the pcDNA3. Transfections were performed using TurboFect (Thermo Scientific, Rockford, lL, USA) according to the manufacturer's instructions.

### Immunocytochemistry

Immunocytochemistry was performed using ibidi *μ*-Slides or *μ*-Dishes (Ibidi, München, Germany) as described.^30^ The cells were fixed by 4% paraformaldehyde (Sigma) in PBS, washed three times with PBS and permeabilized with 0.4% Triton X-100 in PBS. After blocking with 2% BSA in PBS for 30 min, the cells were incubated with appropriate primary antibody at 4 °C overnight. The cells were incubated with FITC or rhodamine B-conjugated secondary antibody and observed using a confocal laser scanning microscope (LSM510 META, Carl Zeiss, Jena, Germany).

### Comet assay

The Comet assay was performed using an OxiSelect Comet Assay Kit (Cell Biolabs, San Diego, CA, USA) as described by manufacturer.^16^ Briefly, cells in agarose slide were lysed, treated with alkaline solution and electrophoresed. The precipitated chromosomal DNA with 70% ethanol were stained with Vista Green DNA Dye and then observed by fluorescent microscopy (Leica DM IL LED, Leica Microsystems, Wetzlar, Germany).

### Co-IP and western blot

For immunopreciptation, cell lysates in RIPA buffer (Millipore, Temecula, CA, USA) were incubated with anti-FLAG M2 affinity gel, anti-HA agarose or anti-c-Myc agarose in IP buffer and centrifuged. Agarose pellet was boiled in 1 × sample buffer. For western blotting, the proteins in cell lysates were separated by SDS-PAGE and transferred to PVDF membrane (Millipore). The membrane was blotted with primary and secondary antibodies conjugated with HRP substrate (Millipore). The enhanced chemiluminescent was detected using chemiluminescence (ECL) kit (Millipore). The band intensity was quantified by AlphaEaseFC software (Alpha Innotech, San Leandro, CA, USA).

### Statistical analysis

All values were shown as the mean±s.d. of one experiment performed in triplicate. Similar results were obtained from at least three independent experiments. Data were analyzed using Student's *t*-test for two group's comparisons or using the Student–Newman–Keuls (SNK) test for multiple comparisons to determine statistical significance (*P<0.05, **P<0.01, ***P<0.005).
