# Supplementary figures and images for: Stability of the cancer target DDIAS is regulated by the CHIP/HSP70 pathway in lung cancer cells
Source: Cell Death Dis. 2017 Jan 12;8(1):e2554–. doi: 10.1038/cddis.2016.488 (PMC5386388; doi:10.1038/cddis.2016.488)

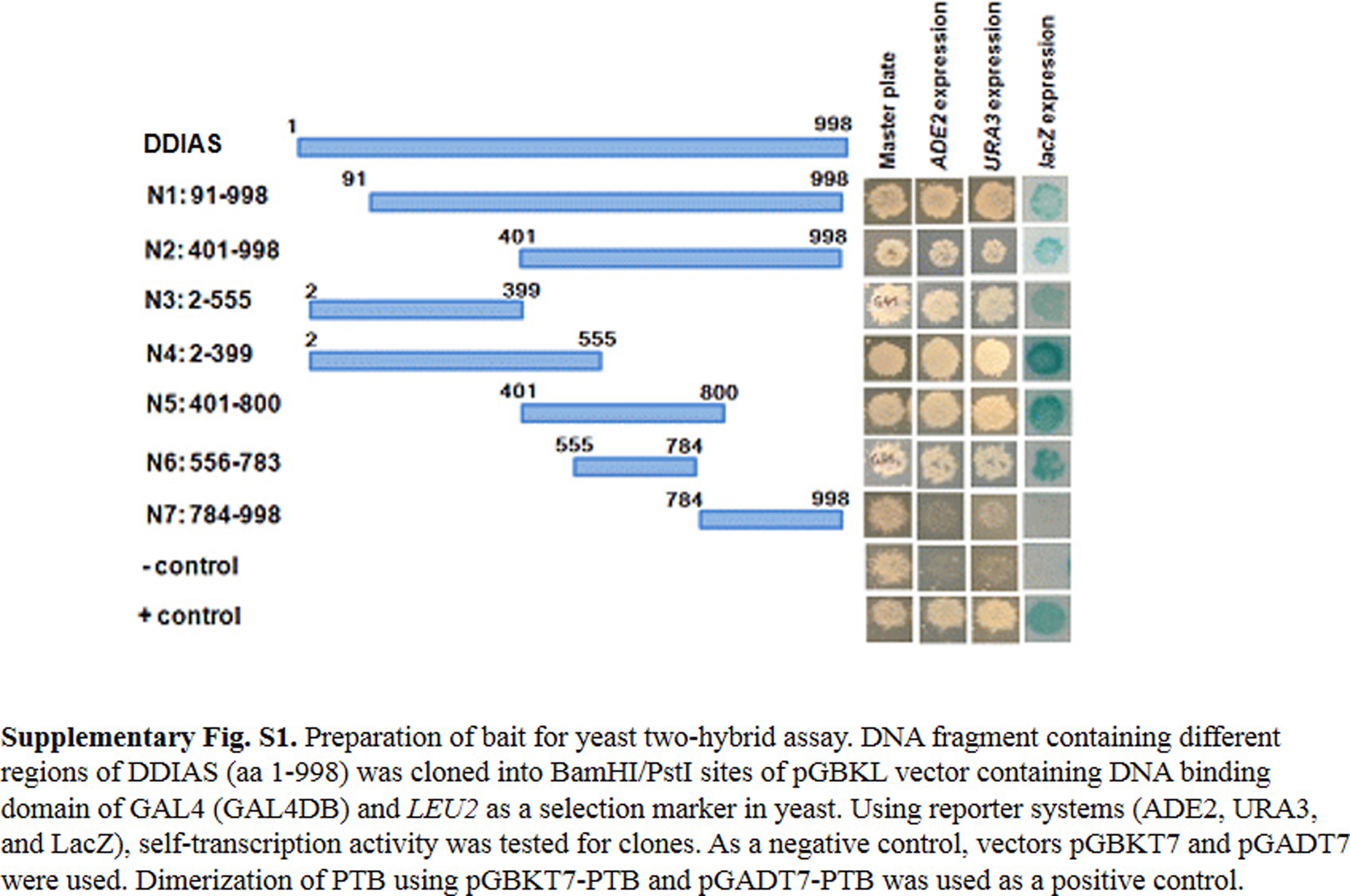

Supplement: Supplementary Figure 1 [file cddis2016488x1.tif]

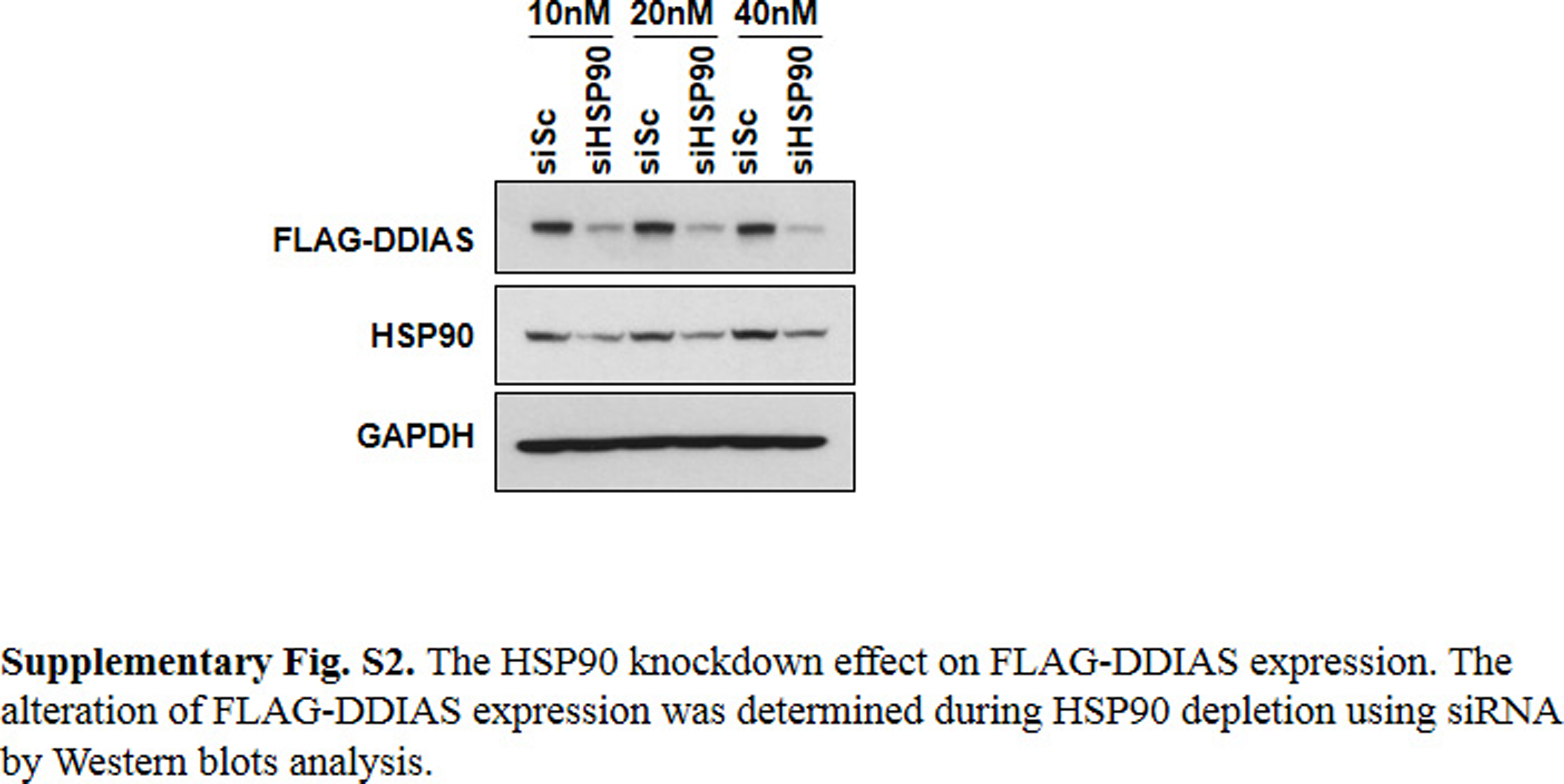

Supplement: Supplementary Figure 2 [file cddis2016488x2.tif]
